# Dexmedetomidine alleviated neuropathic pain in dorsal root ganglion neurons by inhibition of anaerobic glycolysis activity and enhancement of ROS tolerance

**DOI:** 10.1042/BSR20191994

**Published:** 2020-05-05

**Authors:** Peibin Liu, Tufeng Chen, Fang Tan, Jingling Tian, Lei Zheng, Yingqing Deng, Jiaxin Chen, Xinjin Chi

**Affiliations:** 1 Department of Anaesthesiology, The Seventh Affiliated Hospital of Sun Yat-Sen University, Shenzhen 518071, P.R China; 2 Department of Gastrointestinal Surgery, The Third Affiliated Hospital of Sun Yat-Sen University, Guangzhou 510530, P.R China

**Keywords:** anaerobic glycolysis, apoptosis, dexmedetomidine, dorsal root ganglion neurons, neuropathic pain, reactive oxygen species

## Abstract

Neuropathic pain is a kind of chronic pain that is triggered or caused primarily by damage to the nervous system and neurological dysfunction. It’s known that dexmedetomidine is a new type of highly selective alpha2-adrenoceptor agonist with sedation, anti-anxiety, analgesic and other effects. However, the function and mechanism of dexmedetomidine on neuropathic pain are not clear. Rat DRG neurons were isolated and identified using immunofluorescence assay. Following treatment with H_2_O_2_, dexmedetomidine or ROS inhibitor (NAC), the apoptosis and ROS levels were examined by flow cytometery; apoptosis- and anaerobic glycolysis-related proteins were determined by Western blot assay; glucose consumption, pyruvic acid, lactic acid and ATP/ADP ratios were also measured. The results revealed that dexmedetomidine inhibited H_2_O_2_-induced apoptosis and reactive oxygen species (ROS) in rat DRG neurons and in addition, dexmedetomidine down-regulated the expression levels of anaerobic glycolysis-related proteins, significantly reduced glucose, pyruvic acid and lactic acid levels. It also increased the ATP/ADP ratio in H_2_O_2_-treated rat dorsal root ganglion (DRG) neurons. Moreover, we also demonstrated that ROS inhibitor (NAC) also inhibited H_2_O_2_-induced apoptosis and anaerobic glycolysis in rat DRG neurons. In conclusion, dexmedetomidine suppressed H_2_O_2_-induced apoptosis and anaerobic glycolysis activity by inhibiting ROS, in rat DRG neurons. Therefore, dexmedetomidine might play a pivotal role in neuropathic pain by the inhibition of ROS.

## Introduction

Neuropathic pain refers to the douleur anormale (abnormal pain), caused by damage and dysfunction of the central or peripheral nervous system [1,2]. Neuropathic pain is one type of chronic pain, whose main clinical manifestations include hyperalgesia, paresthesia, ambulatory pain and allodynia [3,4]. According to statistics, the prevalence rate is 0.6–1.5% and with the aging of the population, the prevalence rate increases gradually [5]. At present, the treatment of neuropathic pain is mainly through drug therapy, while there are a number of side effects, and the curative effect is low [6]. Currently, only 30–40% of patients have greater than 50% pain relief from medication [7]. This shows that the therapeutic modes of neuropathic pain are not ideal in clinical practice. Therefore, exploring the pathogenesis of neuropathic pain might provide reliable basis and important medical insights into the treatment of pain. There is a growing body of studies indicating that apoptosis of dorsal root ganglion (DRG) neurons is involved in the development of neuropathic pain [8], but its mechanism is still unclear.

In recent years, studies have revealed that reactive oxygen species (ROS) plays a crucial role in the development of neuropathic pain [9,10]. ROS scavengers, such as phenyl-tert-butyl nitrone (PBN), vitamin E and edaravone can alleviate the manifestations of neuropathic pain in animal models [11,12]. Moreover, it has been shown that intrathecal injection of ROS scavenger has the most significant effect [13]. It has been proved that intracellular ROS can activate many signaling pathways and induce cell death [14,15]. Therefore, induction of DRG neurons apoptosis may be avital mechanism for ROS to participate in neuropathic pain [16]. Anaerobic glycolysis is a way for cells to provide energy in response to stressors, such as inflammation and hypoxia [17]. Studies have also demonstrated that in the process of anaerobic glycolysis, a large quantity of ROS could be produced in the cells, and the accumulation of ROS leads to the induction of pain and with advancing age it could lead to oxidative stress and even death [18,19]. However, the specific mechanism of anaerobic glycolysis on neuropathic pain hasn’t been fully elucidated.

Dexmedetomidine is a new type of high selectivity α2-adrenergic agonist, with the characteristics of sedation, analgesia, anti-anxiety, inhibition of sympathetic activity, maintenance of hemodynamic stability and no respiratory inhibition [20]. Currently, dexmedetomidine has been used as a safe and effective drug in clinical practice [21]. In 2009, the US Food and Drug Administration has approved dexmedetomidine to be used for sedation during tracheal intubation and mechanical ventilation in patients undergoing general anesthesia. At present, because of its unique properties, it can be administered before anesthesia, or as a general or local anesthesia adjuvant, and it can also be used in the perioperative period of sedation and analgesia [20]. At present, studies have examined the effects of dexmedetomidine on antioxidant stress, amino acid toxicity and the inhibition ROS synthesis [22–24]. However, there are no studies showing the regulatory effect of dexmedetomidine on anaerobic glycolysis in cells, and whether it has an alleviating effect on pain in DRG neurons.

In the present study, we successfully isolated rat DRG neurons and we explored the influence of dexmedetomidine on H_2_O_2_-induced apoptosis, ROS and anaerobic glycolysis. In addition, we further verified the effects of ROS inhibitor (NAC) on H_2_O_2_-induced apoptosis and anaerobic glycolysis in rat DRG neurons.

## Materials and methods

### Animals

A total of 10 Wistar rats (3 weeks old) were purchased from the Medical Experimental Animal Center of Guangzhou University of Traditional Chinese Medicine. Before the experiments, the purchased rats were kept in a suitable environment for 7 days. In the laboratory, the rats had plenty of food and water, the temperature was kept at 20–25°C, the humidity was kept at 50–60%, and the light was maintained for 12 h every day. The present study was performed with the approval of the Institutional Animal Care and Use Committee of Sun Yat-sen University, China, and in accordance with the principles stated in the Guide for the Care and Use of Laboratory Animals.

### Cell culture

DRG neurons were separated from the Wistar rats. The rats were decapitated after being anesthetized with isoflurane. The spine was removed from the dorsal side and DRGs were collected from the spinal cord and placed in cold F12 medium (Biochrom, Germany). Next, the spinal cord was cut and the capsule was opened under the microscopeand the capsules were incubated in F12 medium (0.9 ml) and collagenase (0.1 ml, 2612.5 U/ml) for 45 min at 37°C with 5% CO_2_. After washing, DRGs were trypsinized and cultured in F12 medium and 10% fetal calf serum (FBS) in an incubator. After 3 days, the cell morphology was observed under an inverted microscope.

### Cell treatment

Rat DRG neurons were treated with 0, 100, 200, 300, 400 and 500 μM dexmedetomidine for 24 h. Following treatment with 100 μM H_2_O_2_ for 4 h [25], rat DRG neurons were treated with dexmedetomidine (1/10 IC50) or 5 mM N-Acetyl-L-cysteine (NAC; Amresco), respectively.

### CCK-8 assay

Cell viability was measured using CCK-8 solution (Dojindo Biochemicals, Kumamoto, Japan). The treated rat DRG neurons (5000/well) were seeded in 96-well culture plates. After incubation for 24 h, 10 μl CCK-8 solution was added to each well. After 2 h, a microplate reader (Bio-Rad, MA, U.S.A.) was used to assess the light absorbance. Following this, the 50% inhibitory concentration (IC50) was calculated. Based on the IC50, we adopted 1/10 IC50 for subsequent experiments.

### Western blotting analysis

RIPA buffer (Beyotime, Shanghai, China) was utilized to extract proteins from the treated DRG neurons. BCA Protein Assay Kit (Thermo, Cat no. 233225) was applied to examine the concentration of protein in each group. The extracted proteins (30 μg) were separated by10% SDS-PAGE and transferred onto polyvinylidene fluoride (PVDF) membranes (Millipore). The membranes were then blocked with 5% non-fat dry milk and incubated with primary antibodies at 4°C overnight. After washing, the membranes were incubated with the secondary antibody (1/3000, Abcam, ab205719). The blots were obtained by using the enhanced chemiluminescence (Thermo Scientific). The primary antibodies contain anti-GAPDH (1/2000, Santa Cruz), anti-NADPHase (1/1000, Abcam, ab127942), anti-HK2 (1/1000, Abcam, ab104836), anti-PFK1 (1/1000, Abcam, ab170868), anti-PK2, (1/1000, Abcam, ab76747), anti-Glut1 (1/1000, Abcam, ab40084), anti-LDHA, (1/1000, Abcam, ab125683), anti-PDK1 (1/1000, Abcam, ab110025), anti-Bax, (1/1000, Abcam, ab53154) and anti-Bcl-2 (1/1000, Abcam, ab196495), antibodies.

### Immunofluorescence (IF) assay

The coverslips were placed into six-well plates, and the treated DRG neurons were seeded on the coverslips. The cells were cultured at 37°C with 5% CO_2_ for 6 h. After washing, the coverslips were fixed with 4% paraformaldehyde (Sigma, Cat# P6148) for 30 min and washed with PBS for three times. Then, cells were ruptured using 50 μl 0.2% Triton X-100 and washed with PBS for three times. The cells were blocked by 1 ml 1% BSA for 1 h and then treated with primary antibody MAP2 (Abcam, ab32454) for 2 h at room temperature and Goat Anti-Mouse IgG H&L (Alexa Fluor® 647, Abcam, ab150115) for 1.5 h at room temperature. After washing, the coverslips and incubated with DAPI (Sigma, cat# NY-S347T) for 10 min. The stained cells were observed under a laser confocal microscope and the images were collected.

### Cell apoptosis assay

Cell apoptosis was evaluated using Annexin V-FITC/PI Apoptosis Kit (Abnova, cat# KA3805). The treated DRG neurons were collected in a 10 ml centrifuge tube and centrifuged (1000×***g*** for 5 min). After washing with PBS, the treated DRG neurons were recollected. The cells were incubated with 5 μl of Annexin V-FITC for 10 min at room temperature in dark. Then, the cells were incubated in 5 μl PI solution at room temperature in dark. The apoptotic cells were assessed using a FACSCalibur Flow Cytometer (Becton Dickinson, San Jose, CA, U.S.A.).

### Flow cytometry for ROS expression

According to previous research [26], the fluorescent dye DHE was used to examine the ROS level. The DRG neurons (1 × 10^6^ cells) were treated with 2.5 mmol/l DHE for 25 min at 37°C. After washing with PBS, cells were collected and stained with red fluorescence dye. Finally, the results were obtained using flow cytometry.

### Glucose measure

Glucose was examined by Glucose Uptake Colorimetric Assay Kit (Elabscience, cat#E-BC-K268). Glucose standards were prepared according to experimental instructions. A total of eight different concentration standards and samples were added to the 96-well plate. The 300 μl working enzyme solution was added to each well, and the 96-well plate was incubated for 15 min at 37°C. The OD values were obtained using a microplate reader at 505 nm. The level of glucose was calculated according to the OD values.

### Pyruvic acid detection

The level of pyruvic acid was confirmed by Pyruvate Assay Kit (Nanjing Jiangcheng Bioengineering Institute, Nanjing, China; cat#A081). Briefly, according to the experimental instructions, the reagents were mixed and incubated for 5 min. The OD values were assessed using a microplate reader at 505 nm and the level of pyruvic acid was analyzed.

### Lactic acid detection

The level of lactic acid was determined by lactic acid assay kit (Nanjing Jiangcheng Bioengineering Institute, Nanjing, China; cat#A019-2). Similarly, following the instructions, all reagents were mixed and incubated for 10 min at 37°C. The OD values were evaluated using a microplate reader at 530 nm. The level of lactic acid was calculated based on the OD values.

### ATP/ADP detection

ATP/ADP ratio was measured by ADP/ATP Ratio Assay Kit (Abnova, cat# KA1673). The treated DRG neurons (1 × 10^4^ cells) were cultured in a microwell plate. ATP reagent was prepared at the following concentration: 95 μl assay buffer, 1 μl substrate, 1 μl co-substrate and 1 μl ATP enzyme. Added 90 μl ATP reagent in each well and incubated for 1 min and the Relative Light Units (RLU A) were obtained. ADP reagent was prepared at the following dilution: 5 μl double steamed water and 1 μl ADP Enzyme and the RLU B were obtained. ATP/ADP = (RLU A)/ ((RLU C) − (RLU B)).

### Statistical analysis

All experiments were repeated three times, the results were displayed as mean  ±  standard deviation (SD), and the statistical analysis was performed using SPSS 18.0 (SPSS Inc., Chicago, IL, U.S.A.) with one-way analysis of variance (ANOVA).

## Results

### Identification of rat DRG neurons

**Figure 1:**
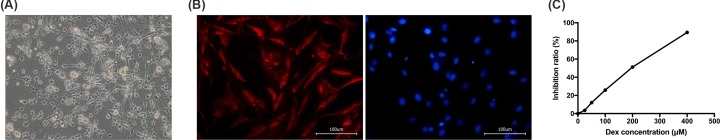
Identification of rat DRG neurons and concentration screening of dexmedetomidine (**A**) Cultured rat DRG neurons were observed using an inverted microscope, magnification, 100×. (**B**) MAP2 expression was elicited by IF assay, in rat DRG neurons, magnification, 100×, scale bar = 100 μm. (**C**) Rat DRG neurons were treated with 0, 100, 200, 300, 400 and 500 μM dexmedetomidine for 24 h. Cell proliferation was determined by CCK-8 assay, and IC50 was calculated.

To study neuropathic pain, we isolated rat DRG neurons. The cellular morphology of DRG neurons was as follows: cells demonstrated a round morphology with large somas and several protuberances and there were also a small number of glial cells and non-neuronal nuclei (Figure 1A). In addition, we used IF assay to examine MAP2 expression in rat DRG neurons, and the results showed that the positive expression rate of MAP2 was more than 80% in rat DRG neurons, suggesting that the effect of cell isolation was good (Figure 1B).

### Concentration screening of dexmedetomidine

To explore the therapeutic effect of dexmedetomidine on neuropathic pain, rat DRG neurons were treated with different concentrations of dexmedetomidine for 24 h and cell proliferation was calculated. The results revealed that dexmedetomidine could inhibit DRG neuron proliferation, and the IC50 of dexmedetomidine was 208.4 μM (Figure 1C). In our subsequent experiments, we treated the rat DRG neurons with 1/10 IC50 (20.84 μM) dexmedetomidine.

### Dexmedetomidine inhibited H_2_O_2_-induced apoptosis and ROS production in rat DRG neurons

**Figure 2:**
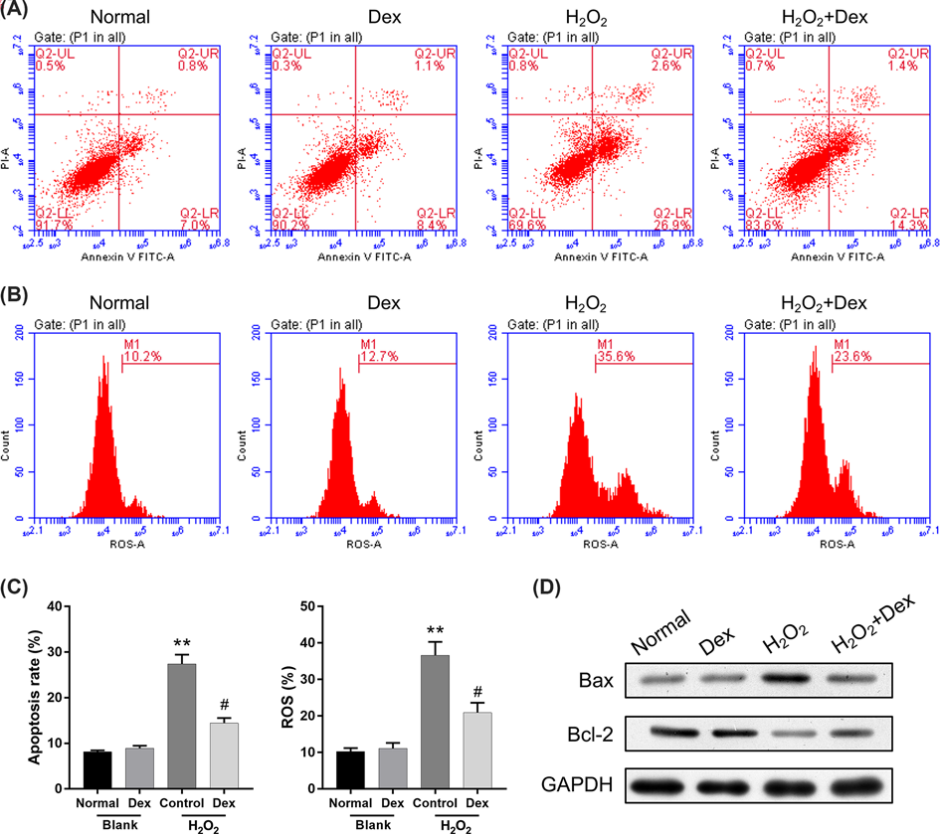
Dexmedetomidine inhibited apoptosis and ROS production in H_2_O_2_-induced rat DRG neurons Rat DRG neurons were treated with dexmedetomidine and H_2_O_2_. (**A**) Annexin V FITC/PI staining was used to determine cell apoptosis. (**B**) The level of ROS was analyzed using a flow cytometer with DCFH-DA fluorescent probe. (**C**) The apoptosis rate and ROS levels were quantitatively calculated (**P<0.01 vs. normal group; #*P*<0.05 vs. Dex group). (**D**) Bax and Bcl-2 expressions were determined by Western blot analysis. GAPDH acted as an internal control.

Following treatment with H_2_O_2_, rat DRG neurons were treated with dexmedetomidine (1/10 IC50, 20.84 μM). Annexin V FITC/PI staining demonstrated that cell apoptosis was dramatically enhanced in the H_2_O_2_-treated group compared with the control group. However, cell apoptosis was markedly reduced in the H_2_O_2_+dexmedetomidine group relative to the H_2_O_2_ group (*P*<0.05, *P*<0.01; Figure 2A,C). Meanwhile, the ROS level was higher in the H_2_O_2_ group compared with the control group; whereas the ROS level was lower in H_2_O_2_+dexmedetomidine group compared with the H_2_O_2_ group (*P*<0.05, *P*<0.01; Figure 2B,C). We also found that H_2_O_2_ up-regulated Bax expression and down-regulated Bcl-2 expression, while dexmedetomidine could reverse Bax and Bcl-2 expressions mediated by H_2_O_2_ in rat DRG neurons (Figure 2D).

### Dexmedetomidine suppressed H_2_O_2_-induced anaerobic glycolysis in rat DRG neurons

**Figure 3:**
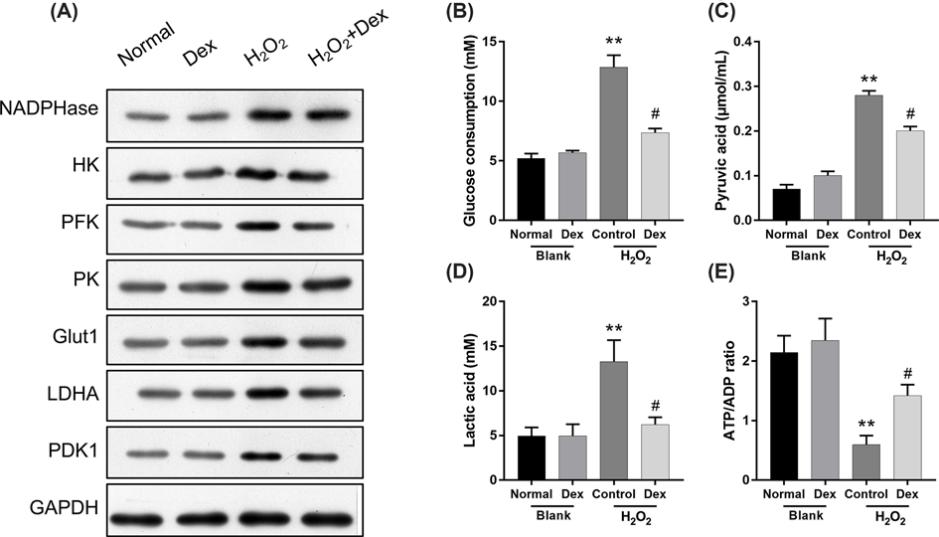
Dexmedetomidine suppressed anaerobic glycolysis in H_2_O_2_-induced rat DRG neurons Dexmedetomidine and H_2_O_2_ were used to treat rat DRG neurons. (**A**) Western blot assay was performed to determine the levels of NADPHase, HK2, PFK1, PK2, Glut1, LDHA and PDK1. GAPDH served as a control for normalization. (**B**) Glucose consumption was analyzed using Glucose Uptake Colorimetric Assay Kit. (**C**) The level of pyruvic acid was measured using Pyruvate Assay Kit. (**D**) The concentration of lactic acid was determined by lactic acid assay kit. (**E**) The ATP/ADP ratio was confirmed by ADP/ATP Ratio Assay Kit. **P<0.01 vs. normal group; #*P*<0.05 vs. H_2_O_2_ group.

To further analyze the possible mechanism of action of dexmedetomidine on neuropathic pain, we investigated the changes of anaerobic glycolysis in rat DRG neurons, induced by H_2_O_2_. The results from Western blot assay revealed that NADPHase, HK2, PFK1, PK2, Glut1, LDHA and PDK1 expressions were markedly increased in the H_2_O_2_ group compared with the control group, whereas NADPHase, HK2, PFK1, PK2, Glut1, LDHA and PDK1 expressions were markedly decreased in the H_2_O_2_+dexmedetomidine group when compared with the H_2_O_2_ group (Figure 3A). In addition, glucose consumption, pyruvic acid and lactic acid expression levels were analyzed, and the results indicated that glucose consumption, pyruvic acid and lactic acid levels were significantly increased in the H_2_O_2_ group compared with the control group, while they were significantly reduced in the H_2_O_2_+dexmedetomidine group in relation to the H_2_O_2_ group (*P*<0.05, *P*<0.01; Figure 3B–D). Simultaneously, we also found that the ATP/ADP ratio was significantly decreased in H_2_O_2_ group compared with the control group, while it was significantly increased in H_2_O_2_+dexmedetomidine group compared with the H_2_O_2_ group (*P*<0.05, *P*<0.01; Figure 3E). Therefore, we determined that dexmedetomidine could inhibit H_2_O_2_-mediated anaerobic glycolysis in rat DRG neurons.

### Dexmedetomidine reduced H_2_O_2_-induced apoptosis by inhibiting ROS in rat DRG neurons

**Figure 4:**
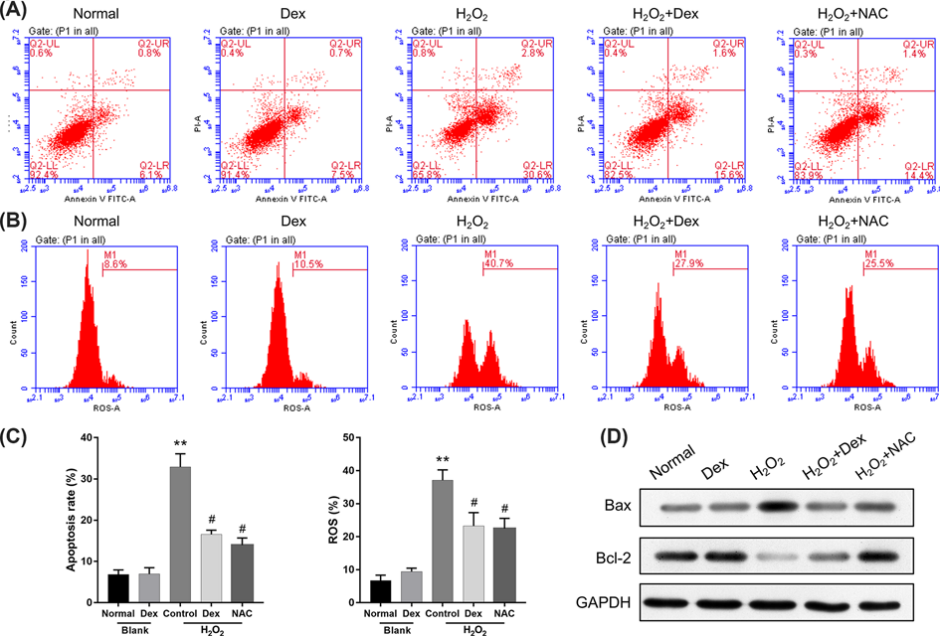
Dexmedetomidine reduced H_2_O_2_-induced apoptosis by inhibiting ROS in rat DRG neurons Rat DRG neurons were treated with H_2_O_2_, dexmedetomidine or/and ROS inhibitor (NAC). (**A**) Cell apoptosis was examined by Annexin V FITC/PI staining. (**B**) Flow cytometer with DCFH-DA fluorescent probe was used to evaluate ROS levels. (**C**) Quantitative analyses of the apoptosis rate and ROS level, **P<0.01 vs. normal group; #*P*<0.05 vs. H_2_O_2_ group. (**D**) The Western blotting analyses of Bax and Bcl-2 expressions.

ROS participates in many cellular metabolic processes, such as growth, proliferation and apoptosis by regulating various signaling pathways [27,28]. To further study the inhibitory effects of dexmedetomidine on H_2_O_2_-induced apoptosis, we adopted ROS inhibitor (N-Acetyl-L-cysteine, NAC) to treat the H_2_O_2_-induced rat DRG neurons. The results of Annexin V FITC/PI staining demonstrated that both dexmedetomidine and NAC significantly inhibited H_2_O_2_-induced cell apoptosis in rat DRG neurons (*P*<0.05, *P*<0.01; Figure 4A,C). We also demonstrated that both dexmedetomidine and NAC significantly reduced H_2_O_2_-induced ROS levels in rat DRG neurons (*P*<0.05, *P*<0.01; Figure 4B,C). In addition, we found that, when compared with H_2_O_2_ group, both dexmedetomidine and NAC dramatically decreased Bax expression, and increased Bcl-2 expression (Figure 4D). Therefore, we determined that dexmedetomidine might be involved in the suppression of ROS in H_2_O_2_-induced apoptosis in rat DRG neurons.

### Dexmedetomidine attenuated anaerobic glycolysis by inhibiting ROS in rat DRG neurons

**Figure 5:**
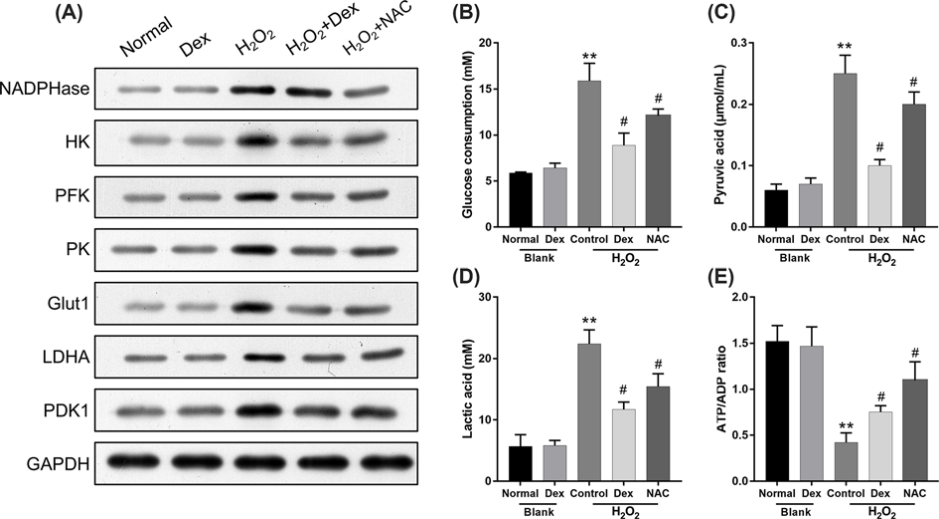
Dexmedetomidine attenuated anaerobic glycolysis by inhibiting ROS in rat DRG neurons Rat DRG neurons were treated with H_2_O_2_, dexmedetomidine and/or NAC. (**A**) Western blotting analysis was utilized to assess the protein levels of NADPHase, HK2, PFK1, PK2, Glut1, LDHA and PDK1 in the treated rat DRG neurons. (**B–E**) Glucose consumption, pyruvic acid and lactic acid expression and ATP/ADP ratios were measured using the corresponding kits; **P<0.01 vs. normal group; #*P*<0.05 vs. H_2_O_2_ group.

Furthermore, we verified the influence and mechanism of dexmedetomidine on H_2_O_2_-mediated anaerobic glycolysis in rat DRG neurons. As shown in Figure 5A, both dexmedetomidine and NAC down-regulated the protein expression levels of NADPHase, HK2, PFK1, PK2, Glut1, LDHA and PDK1, which they were induced by H_2_O_2_ in rat DRG neurons. At the same time, we determined that both dexmedetomidine and NAC could inhibit the levels of glucose consumption, pyruvic acid, lactic acid, which were found to be induced by H_2_O_2_. We also found that dexmedetomidine enhanced the ATP/ADP ratio, which was smothered by H_2_O_2_, in rat DRG neurons (*P*<0.05, *P*<0.01; Figure 5B–E). So, we concluded that the inhibition of anaerobic glycolysis by dexmedetomidine was closely associated with ROS inhibition, in rat DRG neurons.

## Discussion

Peripheral nerve injury often leads to refractory neuropathic pain [2,29]. Studies using animal model have demonstrated that neuropathic pain induced by peripheral nerve injury is often accompanied by apoptosis of spinal DRG neurons [30,31]. A large number of studies have also confirmed that apoptosis of spinal DRG neurons was involved in the process of neuropathic pain [32,33], but the mechanism is still unclear. In our study, we successfully isolated and identified rat DRG neurons, as shown in previous studies [34].

ROS are free radicals produced by organisms, which include active singlet oxygen, hydrogen peroxide and other oxygen free radicals [35]. Once these free radicals are produced, they can be transformed into each other, which will cause oxidative damage to macromolecules, hyperoxidative degeneration, cross-linking or fracture, destruction of cellular structure and function, leading to tissue damage. These injuries have been shown to be the basis of aging, neurodegenerative diseases and tumorigenesis [36]. At present, the removal of excessive ROS has also been considered as an effective treatment for neuropathic pain [37]. The mechanisms of pain relief mainly include increased intracellular Ca^2+^, phosphorylation of NMDA receptors and up-regulation of apoptotic genes, such as bax, caspase-3, caspase-9 and apoptotic protease-activating factor-1 (Apaf-1). In recent years, several studies have suggested that ROS was closely related to a variety of neurodegenerative diseases including amyotropic lateral sclerosis, Parkinson’s disease, Alzheimer’s disease, and injury or aging-related brain dysfunction [38,39]. In addition, a number of studies have revealed that ROS was involved in the apoptosis related to these conditions [40,41]. Other researchers have also demonstrated that low concentrations of H_2_O_2_ can induce apoptosis in a variety of cells [42,43]. In addition, research has also found that dexmedetomidine exhibits an anti-oxidative stress effect by significantly inhibiting the excessive production of ROS. In our study, we further demonstrated that in rat DRG neurons, dexmedetomidine could inhibit H_2_O_2_-induced apoptosis and reduce the level of ROS.

Glycolysis is a process in which glucose or glycogen is degraded to eventually form lactic acid or pyruvate in tissues, while releasing some energy in the form of ATP to be utilized by the tissues [44]. Anaerobic glycolysis refers to the process by which glucose or glycogen decomposes into lactic acid and generates energy, when the body is in a relatively anoxic condition [17]. Therefore, the main indicators of anaerobic glycolysis include glucose consumption, lactic acid and pyruvic acid production and ATP/ADP ratio. These are the markers used in studies of cell anaerobic glycolysis [45,46]. In our study, we found that dexmedetomidine significantly reduced H_2_O_2_-induced glucose consumption, pyruvic acid and lactic acid expression as well as increased the ATP/ADP ratio, in rat DRG neurons. As for the mechanism of action, previous studies have suggested that cells can transport sugars from the extracellular region to the intracellular by glucose transporter (GLUT), which is very important for glucose metabolism [47]. Lactate dehydrogenase A (LDHA) can convert pyruvate into lactic acid, which provides ATP for the body [48]. Pyruvate dehydrogenase 1 (PDK1) is an essential enzyme for pyruvate dehydrogenation by the mitochondria [49]. In addition, several enzymes involved in glycolysis were found to be elevated, such as pyruvate kinase 2 (PK2), phosphofructokinase 1 (PFK1) and hexokinase 2 (HK2), in disease [50]. In our study, we also demonstrated that dexmedetomidine markedly decreased NADPHase, HK2, PFK1, PK2, Glut1, LDHA and PDK1 expression, which were induced by H_2_O_2_, in rat DRG neurons. Therefore, we have shown that dexmedetomidine could dramatically suppress H_2_O_2_-induced anaerobic glycolysis, in rat DRG neurons.

A previous study has indicated that under stress,anaerobic glycolysis of cells can lead to an increase in the production of ROS, and the accumulated ROS can further induce pain [51]. Another study has also demonstrated that inhibition of anaerobic glycolysis could reduce systolic function and mechanical efficiency, while promotion of glycolysis and glucose oxidation could improve the systolic function during ischemia by inhibiting the oxidation of free fatty acids [52]. In our study, we found that treatment with either dexmedetomidine or a ROS inhibitor (NAC) could reduce H_2_O_2_-induced ROS synthesis and apoptosis as well as the levels of glucose consumption, pyruvic acid and lactic acid, in H_2_O_2_-indcued rat DRG neurons. In combination with the inhibitory effect of dexmedetomidine on ROS in H_2_O_2_-induced rat DRG neurons, we further demonstrated that dexmedetomidine could reduce H_2_O_2_-induced apoptosis and anaerobic glycolysis by inhibiting ROS in rat DRG neurons.

In conclusion, our study has demonstrated that dexmedetomidine inhibited H_2_O_2_-induced apoptosis, ROS production andanaerobic glycolysis, similar to a ROS inhibitor (NAC), in rat DRG neurons.

## Abbreviations

Apaf-1: apoptotic protease-activating factor-1
DRG: dorsal root ganglion
HK2: hexokinase 2
LDHA: lactate dehydrogenase A
NAC: N-Acetyl-L-cysteine
PBN: phenyl-tert-butyl nitrone
PDK1: pyruvate dehydrogenase 1
PFK1: phosphofructokinase 1
PK2: pyruvate kinase 2
ROS: reactive oxygen species
